# Phylogenetic Inferences Based on Distinct Molecular Markers Confirm a Novel *Babesia* Species (*Babesia goianiaensis* nov. sp.) in Capybaras (*Hydrochoerus hydrochaeris*) and Associated Ticks

**DOI:** 10.3390/microorganisms11082022

**Published:** 2023-08-06

**Authors:** Felipe da Silva Krawczak, Ana Cláudia Calchi, Lucianne Cardoso Neves, Sarah Alves Dias, Bianca Barbara Fonseca da Silva, Warley Vieira de Freitas Paula, Luiza Gabriella Ferreira de Paula, Mariana Avelar Tavares, Gracielle Teles Pádua, Nicolas Jalowitzki de Lima, Ennya Rafaella Neves Cardoso, Daniel Graziani, Filipe Dantas-Torres, Marcos Rogério André

**Affiliations:** 1 Veterinary and Animal Science School, Federal University of Goiás, Goiânia 74605-220, Brazil; felipekvet@ufg.br (F.d.S.K.); luciannecardoso@discente.ufg.br (L.C.N.); sarah_alves@discente.ufg.br (S.A.D.); biancabarbarafs@gmail.com (B.B.F.d.S.); luizadepaula@ufg.br (L.G.F.d.P.); mariana.tavares@discente.ufg.br (M.A.T.); gracielletelespadua@discente.ufg.br (G.T.P.); jalowitzki@discente.ufg.br (N.J.d.L.); ennyaneves@gmail.com (E.R.N.C.); danielgraziani@ufg.br (D.G.); 2 Vector-Borne Bioagents Laboratory (VBBL), Department of Pathology, Reproduction and One Health, School of Agricultural and Veterinarian Sciences (FCAV), São Paulo State University (UNESP), Jaboticabal 14884-900, Brazil; 3 Laboratory of Immunoparasitology, Department of Immunology, Aggeu Magalhães Institute, Oswaldo Cruz Foundation (Fiocruz), Recife 50740-465, Brazil; filipe.torres@fiocruz.br

**Keywords:** Piroplasmida, *Babesia*, capybara, *Amblyomma*, phylogeny

## Abstract

Piroplasmids (order Piroplasmida) are a diverse group of tick-borne protozoa that may cause disease in animals and occasionally in humans. Novel Piroplasmida clades and species have been found in wild animals from Brazil based on the phylogenetic assessment of near-complete 18S rRNA, mitochondrial and heat-shock protein genes. For instance, a putative novel *Babesia* species has been detected in capybaras and *Amblyomma* ticks in three Brazilian states. The present work aimed to describe, using phylogenetic assessments based on distinct molecular markers, this novel *Babesia* species in capybaras and associated ticks (*Amblyomma sculptum* and *Amblyomma dubitatum*) sampled in Goiânia city, Goiás state, midwestern Brazil. While the phylogenetic analysis based on both near-complete 18S rRNA and *hsp-70* genes positioned the sequences obtained from capybara blood samples into a new clade sister to the *Babesia sensu stricto* clade, the phylogenetic inference based on the COX-3 amino acid positioned the obtained sequences from capybara blood samples and *A. sculptum* ticks also into a clade sister to the *Theileria sensu stricto* clade, highlighting the inappropriateness of this marker inferring evolutionary relationships among piroplasmids. Pairwise distance analysis demonstrated that the divergence rates between the 18S rRNA sequences detected in capybaras and other Piroplasmida already described were very high and ranged from 9.4 to 12.9%. Genotype analysis based on the near-full 18S rRNA sequences of the Piroplasmida detected in capybaras and associated ticks demonstrated the occurrence of high genotype diversity at an intra-species level. In conclusion, phylogenetic analyses based on distinct molecular markers supported the description of *Babesia goianiaensis* nov. sp. in capybaras and associated *Amblyomma* ticks. Additionally, a novel phylogenetic clade, apart from the previously described ones, was described in the present study and contributed to untangling the complex evolutionary history of the Piroplasmida.

## 1. Introduction

Tick-borne diseases are a major concern due to the high diversity of agents involved and the risk they pose to domestic animals and humans, which may eventually become infected with tick-borne agents when they enter tick-infested areas [1]. Among various tick-borne pathogens, protozoa belonging to the genera *Babesia* and *Theileria* (phylum Apicomplexa, order Piroplasmida) parasitize the erythrocytes of mammals worldwide [2].

In North America and Asia, there is a growing concern regarding babesiosis in animals and humans, due to the heavy economic losses to the livestock industry, health consequences to domestic animals and the zoonotic risk posed by some *Babesia* spp. of wild animals (e.g., *Babesia microti* and *Babesia divergens*) [3,4]. Although South America is not considered to be endemic for zoonotic *Babesia* spp., which have not been reported in wild animals so far, the circulation of competent tick vectors for other Piroplasmida species that can parasitize domestic and wild animals emphasizes the need for constant surveillance [1]. In this sense, some studies have pointed out the participation of wild animals, such as the capybara (*Hydrochoerus hydrochaeris*), the largest rodent in the world, in the life cycle of piroplasmids and/or associated ticks, such as *Amblyomma sculptum* and *Amblyomma dubitatum* [5]. Since capybaras are hosts for *A. sculptum* ticks and amplifying hosts for *Rickettsia rickettsii*, the agent of Brazilian spotted fever, understanding the role of these rodents as hosts for other microorganisms is important [6,7].

In southern Brazil, Criado-Fornelio et al. [8] detected a new Piroplasmida 18S rRNA sequence in capybaras. Twelve years later, Gonçalves et al. [9] reported the presence of Piroplasmida partial 18S rRNA sequences, showing >97% identity with the previously reported capybara-associated piroplasmid [8] in a female and a nymph of *A. dubitatum*, collected from a capybara and a black rat (*Rattus rattus*), respectively, in the state of Mato Grosso do Sul. In the state of Goiás, central-western Brazil, Neves et al. [5] reported the presence of capybara-associated *Babesia* sp. DNA in capybaras and two female ticks of *A. sculptum* and *A. dubitatum*. Additionally, large merozoites were found parasitizing the erythrocytes of a capybara with very low parasitemia [5]. Perles et al. [10] molecularly detected the capybara-associated Piroplasmida in *A. sculptum* and *A. dubitatum* collected from coatis (*Nasua nasua*) in the state of Mato Grosso do Sul.

Recently, novel Piroplasmida clades and species have been described in wild animals from Brazil based on the phylogenetic assessment of near-complete 18S rRNA, mitochondrial and heat-shock protein genes [11,12,13]. In the same way, a putative novel *Babesia* sp. in capybaras and *Amblyomma* ticks in three Brazilian states has also been described [5,8,9,10]. In the present work, we used phylogenetic assessments based on distinct molecular markers to describe this novel *Babesia* species, recently reported from capybaras and associated ticks (*A. sculptum* and *A. dubitatum*) in Goiânia city, Goiás state, midwestern Brazil [5].

## 2. Materials and Methods

### 2.1. Study Area and Sample Collection

The blood and tick samples from capybaras used herein originated from the study of Neves et al. [5]; see this reference for more details. In brief, capybaras (*n* = 17) were captured in a corral of approximately 90 m^2^ using as bait sugar cane, corn, corn silage and banana leaves, on the campus of the Federal University of Goiás (16°35′42″ S, 49°16′50″ W, 718 m altitude), Goiânia, Goiás, as previously described [5]. The place where capybaras were captured is located in the Cerrado biome, a tropical savanna ecoregion with two distinct seasons, the rainy season from October to April and the dry season from May to September. The handling of capybaras was performed at night, with the aid of a net catcher. Animals were anesthetized with an intramuscular injection of ketamine (10 mg/kg) plus xylazine (0.5 mg/kg). Capybara EDTA-blood samples were subjected to blood smear examination and DNA extraction (DNeasy^®^ Blood and Tissue Kit, Qiagen, Valencia, CA, USA) [5]. Each capybara was carefully inspected for the presence of ticks for 3 min [5]. After tick morphological identification [5], DNA was extracted using the guanidine isothiocyanate protocol for adults [14] and the boiling protocol for nymphs [15].

This study was authorized by the Chico Mendes Institute for Biodiversity (ICMBio; permit No.: 70679-5) and was approved by the Ethical Committee of Animal Use of the Federal University of Goiás (protocol No.: 092/19).

### 2.2. Molecular Characterization of Piroplasmids 

Capybara blood and tick DNA samples previously positive in the PCR assay targeting a short fragment of the 18S rRNA [5] were herein subjected to PCR assays based on the following genetic markers: near-complete 18S rRNA gene (~1500 bp) [16], *cox-1* (~800 bp) [17], *cox-3* (~600 bp) [18,19], *hsp-70* (~700 bp) [20] and ITS-1 (~450 bp) [21].

PCR assays were performed using 5 μL of the DNA samples in a mixture containing 0.75 U Platinum Taq DNA Polymerase (Invitrogen, Carlsbad, CA, USA), PCR buffer (PCR buffer 10 × 100 nM Tris-HCl, pH 9.0, 500 mM KCl), 0.2 mM deoxynucleotides (dATP, dTTP, dCTP and dGTP) (Invitrogen, Carlsbad, CA, USA), 1.5 mM of magnesium chloride (Invitrogen, Carlsbad, CA, USA), 0.5 μM of each primer (Invitrogen, Carlsbad, CA, USA) and sterile ultrapure water (Invitrogen, Carlsbad, CA, USA) comprising a total volume of 25 μL. In nPCR assays, 1 μL of the amplified product from the first PCR reaction was used as the target DNA in the second reaction. In all PCR assays, a DNA sample obtained from a dog experimentally infected with *Babesia vogeli* (Jaboticabal strain) [22] and sterile ultrapure water were used as positive and negative controls, respectively. 

PCR products were separated using electrophoresis on a 1% agarose gel stained with ethidium bromide (Life Technologies™, Carlsbad, CA, USA) in TEB running buffer pH 8.0 at 100 V/150 mA for 50 min. The gels were examined under ultraviolet light illumination using ChemiDoc MP Imaging System (Bio-Rad, Hercules, CA, USA) and photographed using Image Lab Software v.4.1 (Bio-Rad, Hercules, CA, USA). 

The amplified products that showed high-intensity bands on agarose gel electrophoresis, except those amplified for the 18S rRNA gene, were purified using an ExoSAP-IT PCR Product Cleanup Reagent (Applied Biosystems, Foster City, CA, USA) and sequenced. The sequencing was carried out using the dideoxynucleotide chain termination method [23] at the Human Genome and Stem Cell Research Center, USP, São Paulo, Brazil.

### 2.3. Cloning

In order to obtain the nearly complete fragment of the 18S rRNA gene, the positive samples for this gene were subjected to cloning by pGEM-T Easy (Promega^®^, Madison, WI, USA), according to the manufacturer’s recommendations. Six clones were selected from each positive sample according to the blue/white colony system. The colonies that had the gene fragment of interest confirmed by PCR were submitted to plasmid DNA extraction using the Wizard^®^ Plus SV Minipreps DNA Purification Systems (Promega, Madison, WI, USA). Subsequently, 18S rRNA-containing plasmids were subjected to Sanger sequencing with primers M13 F (5′-CGCCAGGGTTTTCCCAGTCACGAC-3′) and M13 R (5′-GTCATAGCTGTTTCCTGTGTGA-3′) [24], which flank the multiple cloning site of the pGEM-T Easy plasmid. In addition, a pair of internal primers (Primer F: 5′-GACGGGTAACGGGGAATTAG-3′ and Primer R: 5′-AGGACATCTAAGGGCATCAC-3′) designed for the sequences of the present study were also used for sequencing.

### 2.4. BLAST and Phylogenetic Analyses

The sequences obtained were submitted to a quality-screening test using Phred-Phrap software (version 23) [25,26] to evaluate the quality of the electropherograms and to obtain the consensus sequences from the alignment of the sense and antisense sequences. The BLASTn program [27] was used to compare the obtained sequences with those previously deposited in the GenBank database [28]. Sequences saved in FASTA format were aligned with other homologous sequences of each agent retrieved from the database GenBank (National Library of Medicine, Bethesda, MD, USA) using MAFFT (available online on 2 June 2023: https://mafft.cbrc.jp/alignment/server/index.html) [29], and edited via Bioedit v. 7.0.5.3 [30]. W-IQ-Tree web server was used for choosing the evolutionary model following BIC criterion as well as for phylogenetic analysis using the maximum likelihood method (available online on 2 June 2023: http://iqtree.cibiv.univie.ac.at/) [31]. Clade support indices were evaluated through bootstrap analyses of 1000 repetitions. The phylogenetic trees were edited using Treegraph 2.0.56-381 beta software [32].

### 2.5. Genetic Diversity Analyses

A pairwise distance matrix with near-complete 18S rRNA sequences was calculated using the *p*-distance method by MEGA X software (version 11) [33,34].

The genetic diversity analysis for the 18S rRNA gene was performed with the sequences obtained in this study aligned to phylogenetically closer sequences of *Babesia* sp. strain capybara. The alignment was performed using MAFFT (available online on 2 June 2023: https://mafft.cbrc.jp/alignment/server/index.html), as described in the Section 2.4. This alignment was used to calculate the nucleotide diversity (π), the diversity of haplotypes (Dh), the number of haplotypes (h) and the average number of nucleotide differences (K), using DnaSP software (version 5) [35]. The Haplotype Network was constructed in population analysis with Reticulate Trees (popART) software (version 1.7) [36], using the TCS network [37].

## 3. Results

Neves et al. [5] previously published the results regarding the occurrence of piroplasmids in capybaras and associated ticks. In total, 11 capybaras (39.3% [11/28]) and 31 ticks (24.8% [31/125]) were positive in the PCR screening assays for piroplasmids (partial 18S rRNA gene). In order to better characterize the putative novel Piroplasmida species found, PCR assays based on the near-complete fragment of the 18S rRNA gene; *cox-1*, *cox-3* and *hsp-70* genes; and the ITS-1 intergenic region were performed. Of these, only the partial *cox-1* gene was not amplified in the samples analyzed.

**Figure 1:**
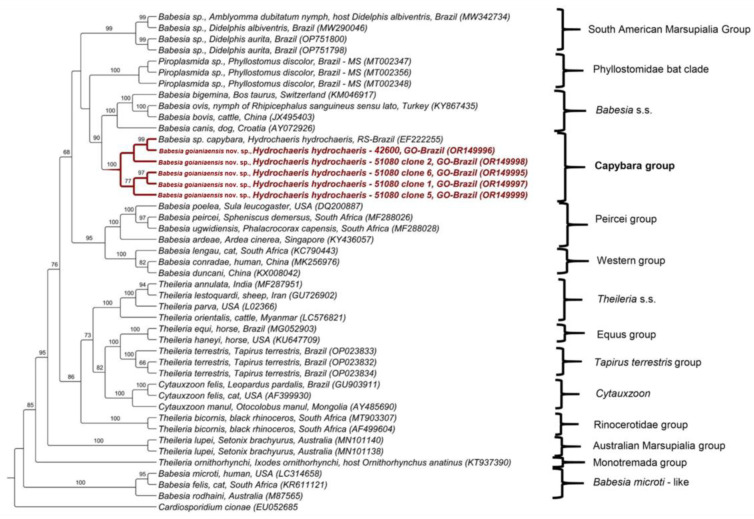
Phylogenetic analysis of piroplasmid 18S rRNA sequences inferred from a 1743 bp alignment generated using maximum likelihood and TIM3 + I + G evolutionary model. Sequences obtained in the present study were highlighted in red. *Cardiosporidium cionae* was used as outgroup.

In order to obtain the near-complete sequence of the 18S rRNA gene, two near-complete fragments of the 18S rRNA gene obtained from capybara blood samples were cloned. As a result, one clone containing the fragment of interest was obtained from one sample (ID 42600), whereas four clones were obtained from another capybara blood sample (ID 51080). The BLASTn analysis demonstrated that the obtained cloned sequences showed identity ranging from 98.29 to 99.30% with a *Babesia* sp. sequence previously detected in the same host in the state of Rio Grande do Sul, Brazil (Table 1). The maximum likelihood phylogenetic analysis performed with a 1743 bp alignment and the TIM3 + I + G evolutionary model positioned the obtained sequences into a new clade sister to the *Babesia sensu stricto* clade, together with the sequence detected in capybara from Rio Grande do Sul, with a bootstrap of 100% (Figure 1). The pairwise distance analysis showed that the divergence among the sequences belonging to this new clade ranged from 0 to 1.67% (Supplementary Material Table S1).

**Figure 2:**
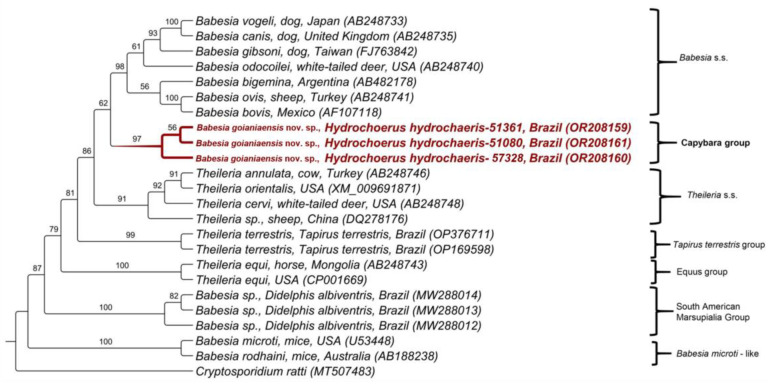
Phylogenetic analysis of piroplasmid *hsp-70* sequences inferred from a 911 bp alignment generated using maximum likelihood and SYM + I + G evolutionary model. Sequences obtained in the present study were highlighted in red. *Cryptosporidium ratti* was used as outgroup.

Regarding the *hsp-70* gene, three sequences amplified from capybara blood DNA samples were obtained. The BLASTn analysis demonstrated that the obtained sequences presented low identity (ranging from 76.40 to 76.85%) with *Theileria orientalis* (Table 1). The phylogenetic analysis by maximum likelihood (the alignment of 911 bp and evolutionary model SYM + I + G) positioned all sequences in the same clade, forming a new clade sister to the *Babesia* sensu stricto clade, with a bootstrap of 97% (Figure 2).

**Figure 3:**
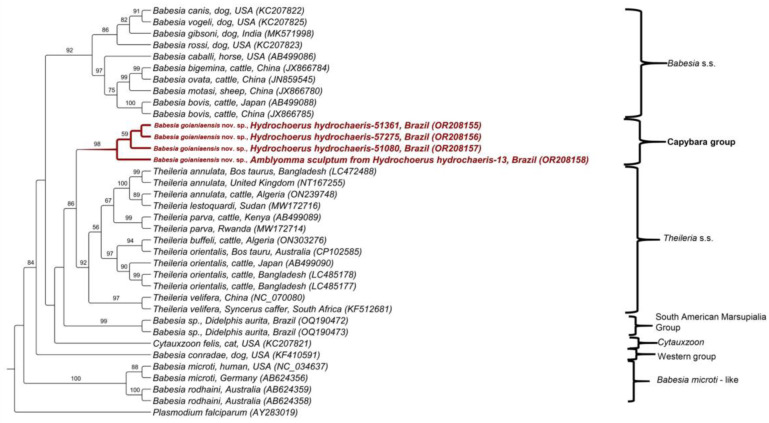
Phylogenetic analysis of piroplasmid *cox-3* protein sequences inferred from a 221 aa alignment generated using maximum likelihood and mtZOA + G evolutionary model. Sequences obtained in the present study were highlighted in red. *Plasmodium falciparum* was used as outgroup.

Four *cox-3* sequences were obtained from three capybara blood samples (ID 51080, 51361 and 57275) and one *A. sculptum* (ID 13 collected from a negative capybara). BLASTn analysis demonstrated that the sequences detected in capybaras presented identity ranging from 73.24 to 76.01% with *T. velifera* (Table 1). The phylogenetic analysis performed with the amino acid (aa) sequences encoded by the *cox-3* gene, with an alignment of 221 aa and evolutionary model mtZOA + G, positioned the sequences into a new clade, close to the *Theileria* sensu stricto clade, with a bootstrap of 98% (Figure 3).

Finally, three intergenic region ITS-1 sequences were obtained: one from a capybara blood sample (ID 41040), one from *A. sculptum* (ID 57) and one from *A. dubitatum* (ID 193). The sequences from capybara blood samples and *A. sculptum* showed identities of 79.8 and 79.63%, respectively, with a sequence of *Theileria* sp. previously detected in *Amblyomma americanum* from the USA. On the other hand, the sequence detected in *A. dubitatum* showed a higher identity (82.82%) with a sequence of *Cytauxzoon felis* detected in a cat from the USA (Table 1). When comparing the three ITS-1 sequences obtained, it was possible to observe that the sequence detected in *A. dubitatum* was different from the others since it presented only 9% of query cover with the other two. In turn, the ITS-1 sequences detected in a capybara and *A. sculptum* showed 99.29% identicalness to each other, with a query cover of 99%.

**Figure 4:**
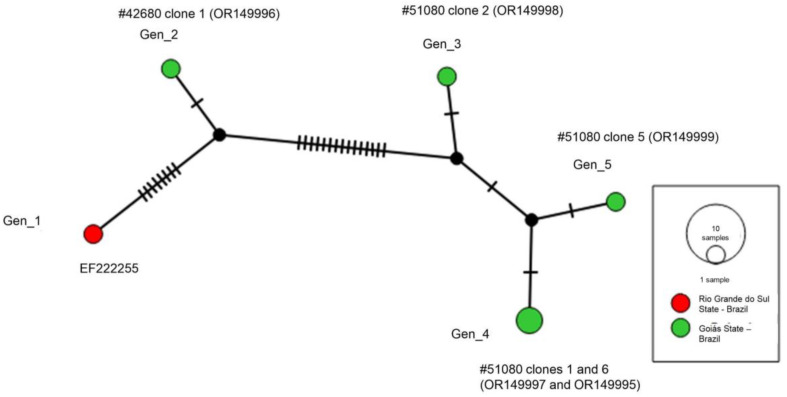
TCS network formed among piroplasmid 18S rRNA sequences (1491 bp) detected in capybaras. The size of the circles varies according to the number of sequences belonging to each genotype, each color represents the location where each sequence was detected, the black vertical lines represent the mutational events that occurred between each genotype and the black circles represent median vectors.

Additionally, a genotype analysis was performed with the near-complete 18S rRNA sequences detected in capybara blood samples (six sequences). The analysis demonstrated the existence of five different genotypes. Genotype #1 comprised the sequence detected in Rio Grande do Sul State, Brazil, while the other genotypes (#2–5) comprised the sequences detected in Goiás State, Brazil. Three different genotypes (#3, #4 and #5) out of four clones obtained were detected in the same animal. Genotype #4 comprised two cloned sequences obtained from the same animal. The analysis showed that all genotypes were derived from median vectors (inferred ancestral nodes) (Figure 4). The nucleotide diversity (π) was 0.00841 ± 0.00245 and haplotype diversity (h) was 0.933 ± 0.122, with 27 variable sites, the average number of nucleotide differences (K) was 12.53333 and 43% of G + C. 

### New Species Description

Family Babesiidae Poche, 1913.

Genus *Babesia* Starcovici, 1893.

*Babesia goianiaensis* nov. sp. Krawczak, Calchi, Dantas-Torres and André.

Type—host: Capybaras (*Hydrochoerus hydrochaeris*) (Mammalia: Rodentia).

Type—locality: Goiânia (16°35′42″ S, 49°16′50″ W, 718 m altitude), Goiás, Brazil.

Other localities: Pelotas (Rio Grande do Sul, southern Brazil) [8] and Campo Grande (Mato Grosso do Sul, midwestern Brazil) [9].

**Figure 5:**
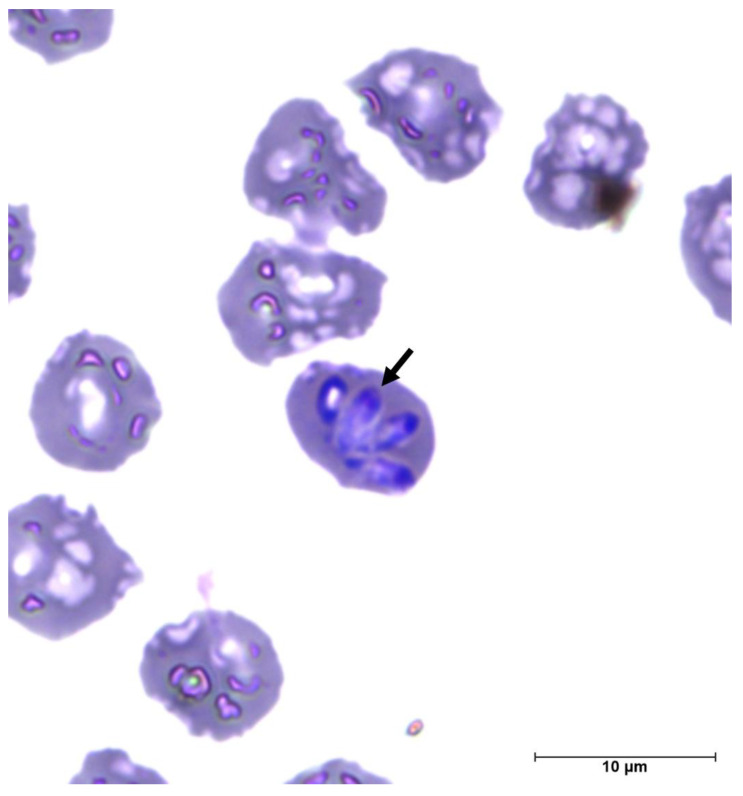
Blood smear of a capybara (*Hydrochoerus hydrochaeris*) presenting merozoites of piroplasmids (black arrow) compatible with *Babesia* (Panótico Rápido^®^ LB, 1000× magnification). The holotype is marked with an arrow.

Type—material: A thin-stained blood smear from a capybara from Goiás State, containing the holotype (Figure 5), was deposited in the Laboratory of Parasitic Diseases (LADOPAR) of the Veterinary and Animal Science School, Federal University of Goiás, under the accession number LAM 001. Moreover, genomic DNA extracted from blood and ticks were also deposited under the accession numbers OR149995–OR149999 for the 18S rRNA gene, OR150000–OR150001 for the ITS-1 region, OR208155–OR208158 for the *cox-3* gene and OR208159–OR208161 for the *hsp-70* gene. 

Vectors: The ticks *Amblyomma sculptum* and *Amblyomma dubitatum* are suspected vectors.

ZooBank registration: To comply with the regulations set out in article 8.5 of the amended 2012 version of the *International Code of Zoological Nomenclature* (ICZN), details of the new species were submitted to ZooBank. The Life Science Identifier (LSID) of the article is urn:lsid:zoobank.org:pub: BC42B530-18A1-4311-9D48-637E77C07BA4. The LSID for the new name *Babesia goianiaensis* nov. sp. is urn:lsid:zoobank.org:act:9F3F512F-6AEB-4AD9-858C-7EB10294CB2B.

Etymology: The new species is named *goianiaensis* in reference to Goiânia city, Goiás state, midwestern Brazil, where the type of material was found.

Description: Merozoites varied in size (Figure 5), appearing pyriform in shape. The mean size of merozoites was 3.9 µm by 1.56 µm (range, 4.12 µm to 3.61 µm by 1.74 µm to 1.43 µm (*n* = 4)). Merozoites pyriform with pale cytoplasm and eccentric, dark-purple-staining nucleus.

## 4. Discussion

In the present study, we formally describe a new species of *Babesia* parasitizing capybaras based on the amplification of the near-complete 18S rRNA gene and different molecular markers (*hsp-70*, *cox-3* and intergenic region ITS-1). More than a decade ago, Criado-Fornelio et al. [8] had already pointed out the occurrence of a possible new species (named *Babesia* sp. capybara) in capybaras sampled in the Rio Grande do Sul state, southern Brazil. A subsequent study reported the presence of this species in Mato Grosso do Sul state, midwestern Brazil [9]. The authors found it in ticks collected from a black rat and from a capybara, but both hosts were negative. Neves et al. [5] found the very same *Babesia* sp. in capybaras and associated ticks in Goiás, midwestern Brazil. More recently, Perles et al. [10] detected this putative novel Piroplasmida in *A. sculptum* and *A. dubitatum* collected from coatis in the state of Mato Grosso do Sul. In the present study, we formally described this species morphologically and phylogenetically, using distinct molecular markers including the near-complete sequence of the 18S rRNA gene.

Morphologically, *Babesia goianiaensis* nov. sp. resembles other large *Babesia* species that infects wild and domestic mammals, thus differing from small *Babesia* species typically found in rodents, such as *Babesia microti* and *Babesia rodhaini*. Congruently, phylogenetic analyses based on the near-complete 18S rRNA gene positioned this new clade as a sister to *Babesia sensu stricto*, which was supported by additional analyses based on the *hsp-70* gene. On the other hand, phylogenetic analysis based on the *cox-3* gene positioned this new clade closer to *Theileria sensu stricto*. This finding could be explained due to the influence that patterns of gene evolution have on phylogenetic positioning [38]. The near-complete 18S rRNA gene has been used as a reliable phylogenetic marker, the conserved nature of which provides high statistical support for clades. Indeed, such a gene is more useful for interspecies differentiation than for intraspecific analysis [39,40,41]. The *hsp-70* gene encodes conserved proteins and has also been considered a good marker for evolutionary studies [42,43]. On the other hand, the *cox-3* gene shows a rapid evolution rate, which can result in low sequence similarities. For instance, Tian et al. [40] showed that there may be differences in the phylogenetic positioning provided by *cox-3* and 18S rRNA due to the rapid evolution and the small fragment size analyzed in the *cox-3*-based phylogenies. Finally, considering that the intergenic region ITS is highly variable, this leads to a complex evolutionary pattern, making it not widely used for phylogenetic analysis [40]. For such a reason, phylogenetic inferences based on the latter molecular marker were not performed in the present study. 

The pairwise distance analysis also supported the definition of a novel Piroplasmida species since the divergence rates between the 18S rRNA sequences detected in capybaras compared to the other piroplasmid sequences already described were very high, ranging from 9.35 to 12.85%. When we compared the divergence among the capybara-associated sequences, lower divergence rates were found among them (range 0 to 1.67%). Noteworthily, distinct Piroplasmida species belonging to the same clade with divergence rates even lower than those found herein have already been reported. For instance, the “Peircei group” is composed of avian-associated *Babesia* species, the 18S rRNA divergence rate of which varies from 0 to 0.44% among *B. poelea*, *B. peircei* and *B. ugwidiensis*. Similarly, Mongruel et al. [12] described a novel *Theileria* species (*Theileria terrestris*) in lowland tapirs (*Tapirus terrestris*) showing low parasitemia from midwestern Brazil, using phylogenetic inferences based on the same molecular markers used herein. Likewise, three novel species of *Cytauxzoon* spp., namely, *C. otrantorum*, *C. banethi* and *C. europaeus*, were described based only on molecular data [44]. These works demonstrate that the diversity of piroplasmids in wild animals is far from being unraveled. Noteworthily, the present work expands the Piroplasmida phylogenetic clades previously proposed by Jalovecka et al. [41], Ikeda et al. [11], Mongruel et al. [12] and Oliveira et al. [13].

Because piroplasmid species cannot be unequivocally distinguished morphologically, phylogenic inferences have been instrumental in the delineation of new species. Nonetheless, there is no well-established consensus about the level of phylogenetic divergence beyond which a species should be regarded as distinct. This may be particularly troublesome when dealing with closely related species that belong to the same clade. The new species delineated herein belong to a clade that does not include any of the known *Babesia* species for which genetic data are available. Future studies on *Babesia* species infecting neotropical rodents and other small mammals will contribute to the understanding of the host range of *Babesia goianiaensis* nov. sp. As more piroplasmid genomes become available, phylogenomics will aid in the resolution of the complex diversity of Piroplasmida, as well as in the definition of criteria for the delineation of novel species. 

The genotype analysis based on the 18S rRNA gene confirmed the occurrence of six different genotypes circulating in capybaras and even in the same animal. Interestingly, three different genotypes (#3, #4 and #5) out of four clones obtained were detected in the same animal. Furthermore, it was possible to observe that genotypes #4 and #5 originated from a single (inferred) ancestral node and were separated by one mutational event. The same pattern was observed for genotypes #1 and #2, albeit there were more mutational events for the formation of the former when compared to the latter. On the other hand, genotype #3 originated from a separate ancestral node. Noteworthily, the analysis showed the formation of two groups: while genotypes #3, #4 and #5 that were detected in the same capybara were closer to each other, genotypes #1 and #2 differed from the first group and were separated by several mutational events. These findings were supported by the phylogenetic inference, as the sequence detected in capybara #42600 (comprising genotype #2) was closer to the sequence detected in capybara from Rio Grande do Sul. The other sequences detected in capybara #51080 were closer to each other, with clone 3 positioned in an intermediate position. The BLASTn analysis also corroborated all these findings, as the sequence that had an identity higher than 99% with the sequence previously detected in Rio Grande do Sul State was obtained from animal #42600, while the other sequences had an identity lower than 99% with this sequence.

Regarding the genotype diversity calculations based on the near-complete 18S rRNA sequences, high genotype diversity (h = 0.933) and low nucleotide diversity (π = 0.00841) were found. Such a divergence is probably due to the low number of sequences evaluated and may indicate small differences between the genotypes. Only 27 variable sites were found, and the average number of nucleotide differences was 12.5. Indeed, these results were expected, considering the following: (i) 18S rRNA is a conserved gene; (ii) the genotype analyses were performed with a small number of sequences; and (iii) some of the cloned sequences were obtained from the same animal. 

In addition to the detection of Piroplasmida sequences in capybaras, the present study also amplified *Babesia* sp. sequences (*cox-3* gene and ITS-1 intergenic region) in *A. sculptum* ticks collected from these animals. Previously, the short 18S rRNA fragment was obtained in this tick species as well as in *A. dubitatum* [5]. Additionally, an *A. dubitatum* tick collected from a capybara sampled in the city of Campo Grande, Mato Grosso do Sul State, was also positive for the small fragment of the 18S rRNA gene. The sequence was positioned in the same clade as the sequences detected in capybaras [9]. This sequence was not added to the phylogeny of the present study due to its small size. This finding leads us to wonder if these tick species could play a role as potential vectors for this new piroplasmid, as discussed elsewhere [5]. In addition to capybaras, horses and tapirs are also the preferred hosts of *A. sculptum* [7]. Considering that all stages of this tick species are considered anthropophilic [7], if this tick species really acts as a vector of *Babesia goianiaensis* nov. sp., there is a chance that this novel Piroplasmida accidentally infects humans and domestic and wild animals. Therefore, future experimental studies to investigate the vector competence of *A. sculptum* and *A. dubitatum* in the transmission of *Babesia goianiaensis* nov. sp. are much needed. Considering that *B. goianiaensis* nov. sp. was positioned into a clade apart from *Babesia* sensu stricto, the importance of transovarian transmission and transstadial perpetuation in the maintenance of this novel Piroplasmida species is yet to be confirmed. Alternatively, the molecular detection of *B. goianiaensis* nov. sp. DNA in ticks alone does not rule out the possibility of *Babesia* sp. DNA presence in ticks due to remnant host blood in both tick species. 

## 5. Conclusions

Phylogenetic analyses based on near-full-length sequences of the 18S rRNA, *hsp-70* and *cox-3* genes supported the description of *Babesia goianiaensis* nov. sp. from capybaras and associated *Amblyomma* ticks from midwestern Brazil. The novel *Babesia* species was positioned apart from other piroplasmids and comprised a sister clade to *Babesia* sensu stricto, as inferred by the near-full-length sequence of the 18S rRNA and *hsp-70* genes. *Babesia goianiaensis* nov. sp. represents a new Piroplasmida clade yet to be characterized regarding biological features, vectors, host specificity and pathogenicity. The presence of different genotypes circulating in the same animal evidences the genetic diversity of this new species.

## Figures and Tables

**Table 1:** BLASTn results of the sequences obtained in the amplification of target genes for piroplasmids in capybaras and associated ectoparasites.

Specie/Identification (GenBank Access Number)	Target Gene	Size (bp)	Query Cover (%)	E-Value	Identity (%)	GenBank Sequence (Access Number)
*Hydrochoerus hydrochaeris*/42600 clone 1 (OR149996)	18S rRNA	1561	100	0	99.30	*Babesia* sp., detected in capybara from Rio Grande do Sul State, Brazil (EF222255)
*Hydrochoerus hydrochaeris*/51080 clone 1 (OR149997)	18S rRNA	1520	100	0	98.29	*Babesia* sp., detected in capybara from Rio Grande do Sul State, Brazil (EF222255)
*Hydrochoerus hydrochaeris*/51080 clone 2 (OR149998)	18S rRNA	1519	100	0	98.42	*Babesia* sp., detected in capybara from Rio Grande do Sul State, Brazil (EF222255)
*Hydrochoerus hydrochaeris*/51080 clone 5 (OR149999)	18S rRNA	1523	100	0	98.29	*Babesia* sp., detected in capybara from Rio Grande do Sul State, Brazil (EF222255)
*Hydrochoerus hydrochaeris*/51080 clone 6 (OR149995)	18S rRNA	1540	100	0	98.31	*Babesia* sp., detected in capybara from Rio Grande do Sul State, Brazil (EF222255)
*Hydrochoerus hydrochaeris*/51080 (OR208161)	*hsp-70*	856	36	1 × 10^−41^	76.85	*Theileria orientalis* (XP_009691871)
*Hydrochoerus hydrochaeris*/51361 (OR208159)	*hsp-70*	877	38	8 × 10^−44^	76.40	*Theileria orientalis* (XP_009691871)
*Hydrochoerus hydrochaeris*/57328 (OR208160)	*hsp-70*	825	40	7 × 10^−44^	76.40	*Theileria orientalis* (XP_009691871)
*Hydrochoerus hydrochaeris*/51080 (OR208157)	*cox-3*	590	84	5 × 10^−34^	73.24	*Theileria velifera*, detected in *Syncerus caffer* from South Africa (KF512681)
*Hydrochoerus hydrochaeris*/51361 (OR208155)	*cox-3*	590	84	5 × 10^−34^	73.24	*Theileria velifera*, detected in *Syncerus caffer* from South Africa (KF512681)
*Hydrochoerus hydrochaeris*/57275 (OR208156)	*cox-3*	590	84	5 × 10^−34^	73.24	*Theileria velifera*, detected in *Syncerus caffer* from South Africa (KF512681)
*Amblyomma sculptum*/13 (OR208158)	*cox-3*	458	78	2 × 10^−37^	76.01	*Theileria velifera*, detected in *Syncerus caffer* from South Africa (KF512681)
*Hydrochoerus hydrochaeris*/41040 (OR150000)	ITS-1 region	572	100	9 × 10^−106^	79.80	*Theileria* sp., detected in *Amblyomma americanum* from USA (KC119627)
*Amblyomma sculptum*/57 (OR150001)	ITS-1 region	568	100	1 × 10^−103^	79.63	*Theileria* sp., detected in *Amblyomma americanum* from USA (KC119627)
*Amblyomma dubitatum*/193 (OR150002)	ITS-1 region	510	100	2 × 10^−116^	82.82	*Cytauxzoon felis*, detected in cat from USA (DQ458797)

## Data Availability

The datasets generated and analyzed during the current study are available on the NCBI GenBank Nucleotide platform (available online on 7 September 2022: https://www.ncbi.nlm.nih.gov/genbank/) and can be accessed through the following accession numbers: OR149995–OR150002; OR208155–OR208161.

## Supplementary Materials

The following supporting information can be downloaded at: https://www.mdpi.com/article/10.3390/microorganisms11082022/s1, Table S1: Distance matrix based on the 18S rRNA gene.
